# Update in Immunotherapy for Advanced Non-Small Cell Lung Cancer: Optimizing Treatment Sequencing and Identifying the Best Choices

**DOI:** 10.3390/cancers15184547

**Published:** 2023-09-13

**Authors:** Katia Roque, Rossana Ruiz, Luis Mas, Daniel Humberto Pozza, Marina Vancini, José Antônio Silva Júnior, Ramon Andrade de Mello

**Affiliations:** 1 Discipline of Medical Oncology, Post-Graduation Programme in Medicine, Faculty of Medicine, Nine of July University (UNINOVE), São Paulo 04101-000, Braziljosejr@uni9.pro.br (J.A.S.J.); 2 Department of Medical Oncology, Instituto Nacional de Enfermedades Neoplásicas, Angamos Este Av., 2520, Lima 15023, Peru; rossana_rm@hotmail.com (R.R.); lmasl@hotmail.com (L.M.); 3 Faculty of Medicine, Universidad Peruana Cayetano Heredia, Lima 15102, Peru; 4 Faculty of Medicine, Universidad Nacional Mayor de San Marcos, Lima 15081, Peru; 5 Escuela Profesional de Medicina Humana-Filial Ica, Universidad Privada San Juan Bautista, Ica 15067, Peru; 6 Department of Medical Oncology, Oncosalud-AUNA, Av. Guardia Civil 571-San Borja, Lima 15036, Peru; 7 Experimental Biology Unit, Department of Biomedicine, Faculty of Medicine of Porto, University of Porto, 4200-319 Porto, Portugal; 8 i3S—Institute for Research and Innovation in Health and IBMC, University of Porto, 4200-319 Porto, Portugal; 9 Oxford Cancer Centre, Oxford University Hospitals NHS Foundation Trust, Oxford OX3 9DU, UK; 10 Department of Oncology, University of Oxford, Oxford OX1 2JD, UK

**Keywords:** immune checkpoint inhibitors (ICIs), non-small cell lung cancer (NSCLC), PD-1, PD-L1, CTLA-4, oncogenic driver mutation, treatment sequencing, tumour biomarkers, personalized medicine

## Abstract

**Simple Summary:**

Immunotherapy stands as a cornerstone in the treatment of advanced lung cancer patients without an oncogenic driver mutation. This comprehensive review provides an overview of immunotherapy treatment options and offers an algorithm for selecting the most appropriate regimen in various clinical scenarios. Additionally, we delve into practical considerations commonly encountered in clinical settings, as well as the latest advancements in the field.

**Abstract:**

The introduction of immunotherapy has brought about a paradigm shift in the management of advanced non-small cell lung cancer (NSCLC). It has not only significantly improved the prognosis of patients but has also become a cornerstone of treatment, particularly in those without oncogenic driver mutations. Immune checkpoint inhibitors (ICIs) play a crucial role in the treatment of lung cancer and can be classified into two main groups: Anti-cytotoxic T lymphocyte antigen-4 (Anti-CTLA-4) and anti-T-cell receptor programmed cell death-1 or its ligand (Anti-PD-1 and Anti-PD-L1). Certainly, the landscape of approved first line immunotherapeutic approaches has expanded to encompass monotherapy, immunotherapy-exclusive protocols, and combinations with chemotherapy. The complexity of decision-making in this realm arises due to the absence of direct prospective comparisons. However, a thorough analysis of the long-term efficacy and safety data derived from pivotal clinical trials can offer valuable insights into optimizing treatment for different patient subsets. Moreover, ongoing research is investigating emerging biomarkers and innovative therapeutic strategies that could potentially refine the current treatment approach even further. In this comprehensive review, our aim is to highlight the latest advances in immunotherapy for advanced NSCLC, including the mechanisms of action, efficacy, safety profiles, and clinical significance of ICI.

## 1. Introduction

The advent of immunotherapy has revolutionized the treatment of advanced non-small cell lung cancer (NSCLC), improving the prognosis of this disease and becoming a key part of the treatment, especially in the population without target mutations [1].

Tumor cells express tumor-specific antigens, which are presented on antigen-presenting cells (APCs), allowing the T cell to recognize the tumor. After that, the CD28 receptor on T cells engages with the B7 receptor (CD80/86) on the APC and activates the T cell [2].

Immune checkpoints are proteins on the surface of T cells and other immune cells that act as negative regulators of immune activity. The first checkpoint to be discovered was Cytotoxic T-lymphocyte antigen-4 (CTLA-4) [1,3]. CTLA-4 competes with CD28 for B7, inducing T-cell cycle arrest and suppressing T-cell activation [4,5]. T-cell receptor programmed cell death-1 (PD-1) is expressed on different immunological cells, including B cells, natural killer cells, and monocytes [5]. PD-1 regulates T-cell and B-lymphocyte activation directly by binding to its ligands PD-L1 and PD-L2 [6]. This complex inhibits the kinase signaling pathway that ordinarily activates T cells [2,6]. Some tumors express PD-L1 and PD-L2, which help to protect against immune response and allow tumor proliferation.

Blocking CTLA-4 and PD-1 [1,7] pathways has proved efficacy in lung cancer. Thus, therapeutic antibodies targeting CTLA-4, PD-1, and its ligand (PD-L1) are now approved as monotherapy and in combination with chemotherapy (Table 1) [1]. Herein, we review the use of immunotherapy for advanced NSCLC.

## 2. Biomarkers

### 2.1. PD-L1

PD-L1 expression as a predictive biomarker of response to PD-1 checkpoint blockade is not a standardized method [1,7]. This is enhanced by the fact that different methods are used to determine the percentage, the different cut-offs, the heterogeneity of the expression, and where it is measured (tumor cells or immune cells) [8]. In this case, the definition of a positive or negative PD-L1 test result depends on the individual antibody, clone, and platform, and is different for each immune checkpoint inhibitor (ICI) [8,9].

Nivolumab trials use 5H1 or 28-8 antibodies developed by Dako, whereas pembrolizumab uses the Dako 22C3 assay. Most of the Atezolizumab trials use Ventana SP263, which measures PD-L1 expression on both tumor cells and immune cells [9,10].

It has not yet been possible to confirm the equivalence between different clones, which would standardize the measurement and make the ICI comparable. The Blueprint PD-L1 IHC Assay Comparison Project evaluated the comparability of four of the different immunohistochemistry assays (22C3, 28-8, SP142, and SP263). The study demonstrated that the percentage of PD-L1-stained tumor cells was comparable between the 22C3, 28-8, and SP263 assays, while the SP142 assay exhibited fewer stained tumor cells. Moreover, data exists which suggest that PD-L1 expression is heterogeneous [11,12,13].

Despite not being the ideal biomarker, PD-L1 expression is currently the best available biomarker to assess whether patients are candidates for ICI. The use of IHC testing for PD-L1 expression is recommended ideally before first-line treatment in all patients with metastatic NSCLC [14]; however, it is not required for prescribing first-line therapy with certain ICI regimens [14,15].

### 2.2. Tumor Mutational Burden (TMB)

TMB is an approximate measure of the total number of somatic mutations per megabase [14,16]. When the number of mutations increases, a high level of neoantigens is generated that activates the response of the immune system against the tumor [17]. Some studies have evaluated the potential use of TMB as a biomarker for deciding whether to use immunotherapy in patients with metastatic NSCLC. However, update data did not find any difference in the response to IO between high and low TMB (≥10 versus <10 mutations per megabase, respectively) [18].

As with PD-L1, there are technical problems with measuring TMB, which include the lack of a standard cut off for high TMB levels, the lack of standard measurements across laboratories and tumors, and the waiting time for the result [15,16]. For these reasons, TMB measurement is not routinely recommended [16].

### 2.3. MAJOR Histocompatibility Complex (MHC)

MHC I and II are vital in immune responses, as they are responsible for presenting antigens and triggering activation of immune cells. MHC I molecules are coded by the genes HLA-A, HLA-B, and HLA-C; and are monitored by CD8+ T cells. Tumor cells employ various mechanisms to decrease MHC-I expression, including HLA gene mutations or deletions, and disruptions in surface transport processes, among other factors [19]. MHC-II, on the other hand, is mainly found on specialized immune cells such as dendritic cells, macrophages, and B lymphocytes. There are varied forms of MHC-II types: HLA-DR, HLA-DP, HLA-DQ. MHC-II expression activates a downstream JAK/STAT pathway and enhances the expression of IFN-response genes [20].

MHC-I loss is documented in approximately a third to half of NSCLC [21]. This is associated with reduced presence of CD8+ tumor-infiltrating lymphocytes (TILs), heightened infiltration of M2-polarized macrophages, and worse prognosis compared to tumors with intact MHC-I expression [22]. MHC-II expression on the tumor surface is observed in a minority of lung cancer cell lines (around 25–33% of NSCLC patients) [21,23]. Ongoing research explores the significance of MHC-II expression on tumor cells, current evidence indicates that it enables the direct presentation of tumor neoantigens to CD4+ T helper cells and is linked to improved outcomes in surgically removed lung adenocarcinomas. A moderate significant correlation exists between higher average HLA gene expression and response to immunotherapy across various cancers; specific data for lung cancer is limited [24]. Further investigations are necessary to understand the role of MHC expression as a predictive biomarker for immunotherapy.

### 2.4. Tumor Microenvironment

The presence of infiltrating CD8+ cytotoxic T lymphocytes consistently represents a key factor in the “hot” immune phenotype. This phenotype signifies an adaptive immune resistance driven by IFN-gamma and is linked to a higher probability of positive tumor responses to ICIs [25].

Some studies evaluated the predictive role of CD8+ T cells for immunotherapy in NSCLC. In practical terms, establishing a predictive measure based solely on CD8+ cell count has proven challenging and lacks consistency as an independent factor [26]. In addition, it is likely influenced by the inherent heterogeneity and limited sample sizes of these studies, along with variations in patient groups, scoring methodologies (immunohistochemistry (IHQ) and mRNA), and in different samples (stroma and tumor, nucleated cell, or proportion of CD8/CD3) [26,27]. Additionally, relying solely on CD8+ cell enumeration might not provide a comprehensive predictive capability, warranting a deeper understanding of the functional state of infiltrating cytotoxic T cells [28,29].

Now, we are going to review the different options of treatment of advanced disease with IO, as single agents or in combination IO-IO or with chemotherapy.

## 3. Chemotherapy Free Regimens

### 3.1. Single Agent

Single agents are recommended as first-line therapy for patients with metastatic NSCLC (mNSCLC) with negative test results for actionable driver mutations and PD-L1 expression levels of 50% or more, regardless of histology. In this setting, we have approvals for pembrolizumab, atezolizumab, and cemiplimab-rwlc (Table 2) [14,15,30].

#### 3.1.1. Pembrolizumab

Pembrolizumab is a humanized IgG4 antibody; it works by blocking the protein PD-1. The phase 3 KEYNOTE-024 study compared single-agent pembrolizumab versus platinum-based chemotherapy (CT) as first-line therapy for patients with mNSCLC with PD-L1 expression levels of 50% or more, without driver mutations and regardless of histology. Its primary endpoint was progression-free survival (PFS).

Patients were administered a fixed dosage of 200 mg of Pembrolizumab every three weeks for a maximum of 35 cycles or were provided platinum-based chemotherapy. Crossover was allowed at disease progression. The pembrolizumab arm exhibited a significantly longer median PFS compared to the CT group (10.3 versus 6.0 months; HR 0.50; 95% CI:0.37–0.68, *p* < 0.001). Likewise, overall survival (OS) at 6 months was 80.2% versus 72.4%, respectively (HR 0.60; 95% CI: 0.41–0.89; *p* = 0.005). The ORR was higher in the pembrolizumab group than in the chemotherapy group (44.8% vs. 27.8%). Treatment-related adverse events (IrAEs) of any grade occurred in 73.4% of patients in the pembrolizumab group, the most common were diarrhea (14.3%), fatigue (10.4%), and pyrexia (10.4%). Only 26.6% of patients presented grade 3 to 5 IrAEs [31].

The 5-year update was published in 2020. With a median follow up of 59.9 months (55.1–68.4), patients in the pembrolizumab arm had a statistically significant benefit in PFS (7.7 versus 5.5 months; HR 0.5; 95% CI: 0.39–0.65) and 5-year overall survival (OS) rate (31.9% versus 16.3%). In addition, PFS2, defined as the time from randomization to subsequent disease progression after initiation of new anticancer therapy or death from any cause, was higher in the pembrolizumab group versus chemotherapy (24.1 versus 8.5 months; HR, 0.51; 95% CI: 0.39–0.67). With these findings, we can conclude that pembrolizumab has a durable and clinically meaningful long-term OS benefit versus chemotherapy as first-line therapy in this population [32].

Single agent pembrolizumab’s role in PD-L1-positve tumors without EGFR mutations or ALK rearrangements was explored in the KEYNOTE-042. This phase 3 RCT compared single-agent pembrolizumab versus platinum-based CT as first-line therapy for patients with untreated advanced NSCLC, regardless of histology.

The main outcomes of the study were OS among patients categorized by tumor proportion score (TPS) of 50% or higher, 20% or higher, and 1% or higher. After a median follow-up period of 12.8 months, the pembrolizumab group demonstrated a statistically significant increase in OS across all three TPS populations (≥50% HR 0.69, 95% CI: 0.56–0.85, *p =* 0.0003; ≥20% HR 0.77, CI: 0.64–0.92, *p* = 0.0020, and ≥1% HR 0.81, CI: 0.71–0.93, *p* = 0.0018). The median OS was 20.0 months for pembrolizumab versus 12.2 months for chemotherapy, 17.7 months versus 13.0 months, and 16.7 months versus 12.1 months, among the patients who had a TPS equal to or higher than 50%, 20%, and 1%, respectively. IrAEs or worse were experienced by 18% (113 out of 636) of those who received pembrolizumab [32,33].

The 5-year update results from this trial, with a median follow-up time of 61.1 months (50.0–76.3), consistently report an OS benefit in all three groups (TPS ≥ 50%, HR 0.68 [0.57 to 0.81]; TPS ≥ 20%, HR 0.75 [0.64 to 0.87]; TPS ≥ 1%, HR 0.79 [0.70 to 0.89]). The 5-year OS rates were 21.9%, 19.4%, and 16.6%, in each group, respectively. Subgroup analysis showed no benefit of this regimen in patients with PD-L1 1–49%, suggesting that the benefit in the PD-L1+ population is driven by the high expressors; thus, although this regimen is approved by the FDA, it is not generally used. No new toxicities were reported [32,33].

#### 3.1.2. Atezolizumab

Atezolizumab is a humanized IgG1 monoclonal antibody that targets PD-L1. Based on results of IMpower 110, the FDA approved in 2021 the use of atezolizumab for patients with mNSCLC, PD-L1 high expresses, squamous and non-squamous histology, and negative test results for actionable driver mutations; as a first-line therapy option [2,6]. The IMpower 110, a phase 3 randomized clinical trial (RCT), compared first-line therapy with single-agent atezolizumab versus platinum-based chemotherapy (CT) in patients with untreated PD-L1 ≥ 1% (on tumor cells or tumor-infiltrating immune cells as assessed by the SP142 IHC assay) mNSCLC. The primary endpoint was OS. In the experimental group, patients received first-line atezolizumab monotherapy, in the control group, patients received platinum (cisplatin or carboplatin)/pemetrexed for non-squamous histology and cisplatin/gemcitabine for squamous histology. Atezolizumab significantly prolonged OS (20.2 versus 13.1 months; HR 0.59; 95% CI: 0.40–0.89, *p* = 0.0106) only in patients with high PD-L1 expression (PD-L1 ≥ 50% of tumor cells or PD-L1 stained tumor-infiltrating immune cells covering ≥ 10% of the tumor area) [34]. An update after 3 years of follow-up confirmed the OS benefit in this population (20.2 versus 14.7 months; HR 0.76, 95% CI: 0.54–1.09) [35]. Despite these excellent results, we should emphasize that in this study, crossover from the chemotherapy arm to the atezolizumab arm was not permitted. IrAEs occurred in 90.2% of patients in the atezolizumab group. Grade 3 to 4 IrAEs occurred in 12.9% of patients receiving atezolizumab monotherapy versus 44.1% with chemotherapy. The most frequent serious adverse reactions with atezolizumab were pneumonia (2.8%), COPD (2.1%), and pneumonitis (2.1%); 28% of patients had serious adverse reactions [34].

#### 3.1.3. Cemiplimab

Cemiplimab is a fully human immunoglobulin IgG4 monoclonal antibody that targets PD-1 [36]. FDA approved its use on the basis of the phase 3 RCT EMPOWER-Lung 1 results. Single-agent cemiplimab showed more effectiveness than platinum-based CT for patients with metastatic NSCLC with PD-L1 levels of 50% or more, without driver mutation and regardless of histology. The primary endpoints were OS and progression-free survival (PFS). Crossover from CT to cemiplimab was allowed. In the PD-L1 ≥ 50% population (n = 563), median OS was not reached in the cemiplimab group versus 14.2 months for the CT group (HR 0.57; 95% CI: 0.42–0.77, *p* = 0.0002) Median PFS was significantly improved in the cemiplimab group versus the CT group (8.2 versus 5.7 months; HR 0.54; 95% CI: 0.43–0.68, *p* < 0.0001). Grade 3 to 4 IrAEs were reported in 28% (98/355) of the cemiplimab group and treatment-related deaths occurred in 2.5% (9/355) of patients; due to autoimmune myocarditis, cardiac failure, cardiopulmonary failure, cardiorespiratory arrest, nephritis, respiratory failure, septic shock, tumor hyper progression, and unknown [37]. An update after 3 years of follow up showed the sustained improvement in OS, PFS, and objective response rate (ORR), despite a 75% crossover rate. In the cemiplimab group, the median OS was 26.1 versus 13.3 months in the CT group (HR 0.57; 95% CI: 0.46–0.71, *p* < 0.0001); median PFS was 8.1 versus 5.3 months (HR 0.51; 95% CI: 0.42–0.62, *p* < 0.0001) and ORR was 46.5% versus 21.0% (OR 3.26; *p* < 0.0001). In addition, crossover patients obtained durable responses and an ORR of 31.3% [38].

## 4. Immunotherapy Combinations

### 4.1. Nivolumab plus Ipilimumab

Nivolumab is a fully human immunoglobulin G4 (IgG4) monoclonal antibody against PD-1 and ipilimumab is an anti-CTLA4. The combination of both demonstrated efficacy in metastatic melanoma, improving ORR and survival compared with monotherapy [36]. These encouraging results prompted its study in lung cancer.

CheckMate 227 compared nivolumab plus ipilimumab, nivolumab monotherapy, and chemotherapy in patients with untreated metastatic PD-L1+ non-squamous or squamous NSCLC. This trial included patients with PD-L1 expression levels ≥ 1% or more and less than 1%, PS 0 to 1, and without driver mutations. The primary endpoint was OS with nivolumab plus ipilimumab as compared with CT in PDL-1 positive. In addition, a co-primary analysis was performed in patients who had high TMB levels (≥10 mutations/megabase). The median OS was 17.1 months with nivolumab plus ipilimumab and 14.9 months with chemotherapy (*p =* 0.007), with 2-year OS rates of 40.0% and 32.8%, respectively. The OS benefit was also observed in patients with a PD-L1 expression less than 1%, with a median duration of 17.2 months vs. 12.2 months, in the nivolumab plus ipilimumab and chemotherapy groups, respectively. Grade 3 to 4 IrAEs were 32.8% in patients who received nivolumab plus ipilimumab. The most common toxicity of any grade were skin reactions (34.0%) and endocrine events (23.8%) [39].

The 5-year updated data showed that OS was improved regardless of TMB or PD-L1 expression levels with nivolumab plus ipilimumab in comparison with CT. In patients with PD-L1 ≥ 1%, the OS rate was 24% for nivolumab plus ipilimumab versus 14% for CT. Furthermore, in patients with PD-L1 < 1%, the OS rates were 19% versus 7% in each group, respectively. Quality of life in 5-year survivors was similar to the general US population through the 5-year follow-up [39,40].

### 4.2. Durvalumab plus Tremelimumab

The MYSTIC trial was a phase 3 RCT designed to compare durvalumab plus tremelimumab, durvalumab alone, and CT in patients with treatment-naïve mNSCLC without sensitizing EGFR or ALK genetic alterations. Patients were randomly assigned in a 1:1:1 ratio to one of the following treatment groups: durvalumab alone (20 mg/kg every 4 weeks), durvalumab (20 mg/kg every 4 weeks) in combination with tremelimumab (1 mg/kg every 4 weeks, for up to 4 doses), or platinum-based doublet CT.

The primary endpoints were OS for durvalumab vs. chemotherapy, OS and PFS for durvalumab plus tremelimumab vs. chemotherapy assessed in patients with ≥25% PD-L1 expression. The median OS was higher for patients who received durvalumab compared with CT, but was not significant (HR 0.76; 97.5% CI: 0.56–1.02; *p* = 0.04). For the group who received durvalumab plus tremelimumab, the median OS was higher than CT, but also was not significant (HR 0.85; 98.77% CI:0.61–1.17; *p* = 0.20). In addition, no significant differences were found in PFS for the durvalumab plus tremelimumab arm versus the CT arm (HR 1.05; 99.5% CI: 0.72–1.53; *p =* 0.71). Although the trial did not achieve its primary endpoints, further exploratory analyses revealed that a threshold of ≥20 mutations per megabase in TMB was associated with higher OS benefit when using the combination of durvalumab and tremelimumab [41].

## 5. Combinations with Chemotherapy

In the previous section, we summarized the different options of single-agent immunotherapy and immunotherapy combinations. On the other hand, different combinations of immunotherapy plus chemotherapy were approved for patients with mNSCLC (Table 3).

### 5.1. Atezolizumab

IMpower150, a phase 3 RCT, evaluated the efficacy of atezolizumab in three groups in metastatic non-squamous NSCLC. Intention to treat (ITT) are wild-type (WT) EGFR and ALK patients; however, patients with EGFR or ALK genomic alterations were considered if they had disease progression or several adverse reactions to tyrosine kinase inhibitors in the past. The first group received a combination of atezolizumab, carboplatin, and paclitaxel (ACP); the second group received a combination of bevacizumab, carboplatin, and paclitaxel (BCP); and the third group received a combination of atezolizumab, bevacizumab, carboplatin, and paclitaxel (ABCP) every three weeks for a total of four to six cycles, followed by maintenance therapy using atezolizumab, bevacizumab, or both. The main objectives were PFS in patients with high expression of an effector T cell (Teff) gene signature within the tumor and OS in the WT population. The median PFS was longer in the ABCP group than in the BCP group (8.3 versus 6.8 months; HR 0.62; 95% CI: 0.52–0.74; *p* < 0.001) and median OS was 19.2 months in the ABCP group and 14.7 months in the BCP group (HR 0.78; 95% CI: 0.64–0.96, *p* = 0.02) [42].

This trial also enrolled some patients with EGFR mutations or ALK rearrangements (n = 108) and disease progression on prior TKI. In this population, the initial results showed a greater PFS benefit with ABCP than with BCP (9.7 versus 6.1 months; HR 0.59; 95% CI: 0.37–0.94) [42]. Nevertheless, the 3y update demonstrated that the benefit in OS was not statistically significant for the ABCP group versus BCP in sensitizing EGFR mutations (HR 0.60; 95% CI: 0.31–1.14) or in a previous TKI population (HR 0.74; 95% CI: 0.38–1.46) [43].

In 2021, an update with a median follow up of 39.8 months was published. In the final analysis, there were no statistically significant improvements in OS between ACP vs. BCP (19.0 versus 14.7 months; HR 0.84; 95% CI: 0.71–1.00). An OS benefit was sustained with ABCP versus BCP (19.5 versus 14.7 months; HR 0.80; 95% CI: 0.67–0.95). In SP142-defined PD-L1 high and PD-L1-positive subgroups, the median OS was longer with ABCP and ACP versus BCP; while in the PD-L1 negative subgroup, there were no differences in OS between ACP and ABCP vs. BCP [44].

IMpower130, a multicenter phase 3 RCT, evaluated the efficacy of atezolizumab plus carboplatin plus nanoparticle albumin-bound (nab)-paclitaxel versus chemotherapy in patients with metastatic non-squamous NSCLC without EGFR mutations or ALK rearrangements, according of PD-L1 tumor expression. The primary endpoints were PFS and OS in the intention-to-treat population. The median OS was 18.6 versus 13.9 months (HR 0.79; 95% CI: 0.64–0.98; *p* = 0.033) and median PFS was 7.0 versus 5.5 months (HR 0.64; 95% CI: 0.54–0.77, *p* < 0.0001) in the atezolizumab plus CT group and in the CT group, respectively. Regarding IrAEs, the most common grade 3 or worse were neutropenia (32% versus 28%) and anaemia (29% versus 20%); treatment-related deaths were reported in 2% (8/473) of patients in the atezolizumab plus chemotherapy arm and in less than 1% (1/232) of patients in the CT only arm [45].

IMpower 131, a multicenter phase 3 trial, included patients with metastatic squamous NSCLC without EGFR mutations or ALK rearrangements regardless of PD-L1 status, divided in 1:1:1 arm to receive atezolizumab + carboplatin + paclitaxel (A + CP), atezolizumab + carboplatin + nab-paclitaxel (A + CnP), or carboplatin + nab-paclitaxel (CnP). The primary endpoints were PFS and OS. PFS was improved in A + CnP versus CnP (6.3 versus 5.6; HR 0.71, 95% CI: 0.60–0.85; *p* = 0.0001). However, the OS had not reached a significant benefit (14.2 versus 13.5; HR 0.88, 95% CI: 0.73–1.05; *p* = 0.16). OS improvement with A + CnP versus CnP was observed in the PD-L1-high subgroup (HR = 0.48, 95% CI: 0.29–0.81), despite not being formally tested. IrAEs grade 3 and 4 occurred in 68.0% and 47.9% (A + CnP) and 57.5% and 28.7% (CnP) of patients, respectively [46].

IMpower132 evaluated the use of atezolizumab + platinum-based chemotherapy + pemetrexed at first line in patients with metastatic NSCLC. Patients were enrolled regardless of PD-L1 expression and randomized in two arms: carboplatin/cisplatin + pemetrexed +/− atezolizumab; following maintenance with pemetrexed +/− atezolizumab. The primary endpoints were PFS and OS. At the primary analysis, the median follow-up was 14.8 months; PFS exhibited significant improvement in patients who received atezolizumab versus only chemotherapy (7.6 versus 5.2 months; HR 0.60, 95% CI: 0.49–0.72, *p* < 0.0001). OS was not statistically significant at the interim analysis (18.1 versus 13.6 months; HR 0.81, 95% CI: 0.64–1.03, *p* = 0.0797). Grade 3 or 4 TRAE (treatment related adverse effect) occurred in 54.6% of patients who received atezolizumab [45,46,47].

### 5.2. Cemiplimab

EMPOWER-Lung 3 evaluated the efficacy of cemiplimab plus platinum-doublet chemotherapy as first-line treatment for a NSCLC, regardless of PD-L1 expression or histology. This study included stage III/IV NSCLC without a driver mutation (EGFR, ALK or ROS1). The primary endpoint was OS. After 16.4 months of follow-up, median OS was 21.9 months with cemiplimab plus chemotherapy versus 13.0 months with placebo plus chemotherapy (HR 0.71; 95% CI, 0.53–0.93; *p* = 0.014). Grade ≥ 3 adverse events occurred in 43.6% of patients who received cemiplimab plus chemotherapy. Immune-related adverse events occurred in 19% and one patient died from immune-mediated pneumonitis [48].

In March 2023, a 2-year update data was published. After 28.4 months of median follow-up, cemiplimab plus chemotherapy significantly improved median OS compared to chemotherapy alone (21.1 versus 12.9 months; HR 0.65, 95% CI: 0.51–0.82, *p =* 0.0003); median PFS was 8.2 months versus 5.5 months (HR 0.55, 95% CI: 0.44–0.68, *p* < 0.0001), and ORRs were 43.6% versus 22.1%, respectively. Safety was similar to the previous report (48.7%) and no immune-related deaths were reported [48,49].

### 5.3. Pembrolizumab

KEYNOTE-189, compared platinum (carboplatin or cisplatin) plus pemetrexed +/− pembrolizumab in patients with untreated metastatic non-squamous NSCLC without EGFR mutations or ALK rearrangements. Patients received doublet chemotherapy plus either 200 mg of pembrolizumab or placebo every 3 weeks for 4 cycles, followed by pembrolizumab or placebo plus pemetrexed for up to a total of 35 cycles as maintenance therapy. The primary endpoints were OS and PFS. Crossover to pembrolizumab monotherapy was permitted. After a median follow-up of 10.5 months, OS at 12 months in the experimental group was better than that of the control group (69.2% versus 49.4%; HR 0.49; 95% CI: 0.38–0.64; *p* < 0.001), regardless of PD-L1 categories. The median PFS was 8.8 versus 4.9 months (HR 0.52; 95% CI: 0.43–0.64; *p* < 0.001). Adverse events of grade 3 or higher occurred in 67.2% of the patients who received pembrolizumab and were similar in both arms [50].

At ASCO 2022, 5-year updates were presented. With a median follow up of 64.6 months (60.1–72.4), patients who received pembrolizumab had a consistent benefit in OS (22 versus 10.6 months; HR 0.6; 95% CI: 0.50–0.72) and PFS (9 versus 4.9 months; HR 0.5; 95% CI: 0.42–0.60). 5-year OS rates were 19.4% versus 11.3%, respectively. Among 57 patients who completed 35 cycles of pembrolizumab, the ORR was 86.0% and the 3-year OS rate after completion of 35 cycles of pembrolizumab was 71.9%. The effective crossover rate was 40.8%. Grade 3–5 IrAEs occurred in 72.8% (295/405 patients). These data confirmed and supported the benefit of pembrolizumab plus chemotherapy in these patients, regardless of PD-L1 status [51].

For metastatic squamous cell NSCLC patients, KEYNOTE-407, a phase 3 RCT, compared carboplatin plus paclitaxel or nab-paclitaxel +/− pembrolizumab, regardless of the level of PD-L1 expression. Patients received carboplatin in combination with either paclitaxel or nab-paclitaxel for the initial four cycles, following 200 mg of pembrolizumab or placebo, for a maximum of 35 cycles. The primary endpoints were OS and PFS. After a median follow-up of 7.8 months, the median OS was 15.9 months and 11.3 months in the pembrolizumab and the placebo group, respectively (HR 0.64; 95% CI: 0.49–0.85, *p* < 0.001). PFS was higher in patients who received CT + pembrolizumab versus CT + placebo (6.4 versus 4.8 months; HR 56; 95% CI: 0.45–0.70, *p* < 0.001). Grade 3 or higher IrAEs were reported in 69.8% of patients in the pembrolizumab-combination group. Furthermore, the rate of treatment discontinuation was higher in this group compared to the placebo-CT group (13.3% versus 6.4%) [52].

In 2022, 5-year efficacy and safety reported a median time from random assignment to data cutoff of 56.9 (49.9–66.2) months. A total of 127/281 patients crossed over from the placebo to the pembrolizumab group, and an additional 26 patients received subsequent anti–PD-(L)1 therapy, with an effective crossover rate of 51.1%. The median OS was 17.2 versus 11.6 months, for the pembrolizumab + CT and placebo + CT group (HR 0.71; 95% CI: 0.59–0.85) and the 5-year OS rates were 18.4% and 9.7%, respectively. The median PFS was higher in the pembrolizumab versus placebo group (8 versus 5.1 months; HR 0.62; CI: 0.52–0.74). In the group of 55 patients who completed 35 cycles of pembrolizumab, ORR was 90.9%, and 3-y OS rate after completion of 35 cycles was 69.5%. Grade 3 to 5 TRAE occurred in 74.8% and 70.0% of patients in each group, respectively.

These findings confirm the previous results, and maintain pembrolizumab in combination with CT as standard-of-care first-line treatment option for metastatic squamous NSCLC, irrespective of PD-L1 [53].

In addition, a pooled analysis of three randomized trials (KEYNOTE-407, KEYNOTE-189, and KEYNOTE-021 cohort G) in patients with advanced/metastatic NSCLC and PD-L1 levels less than 1%. Among 33.4% (444/1328) of patients had PD-L1 negative tumors, OS (HR, 0.63; 95% CI, 0.50–0.79) and PFS (HR 0.68; 95% CI: 0.56–0.83) was improved in patients who received pembrolizumab plus CT versus CT alone. In patients who completed 35 courses of pembrolizumab (n = 16), ORR was 87.5% (95% CI, 61.7–98.4%), and the 3-year OS rate was 100%. TRAE were presented by 99.2% and 98.9%, with grade 3 or higher in 71.4% and 72.0% of the patients who received pembrolizumab plus CT and CT alone, respectively. Immune-mediated adverse effects were experienced by 29% [54].

### 5.4. Nivolumab/Ipilimumab

CheckMate 9LA, a phase 3 RCT, compared first-line nivolumab/ipilimumab and 2 cycles of platinum-doublet induction chemotherapy versus 4 cycles of chemotherapy alone in patients with metastatic NSCLC without driver mutations, regardless of PD-L1 expression levels and histology. Patients were randomly assigned and stratified by tumor histology, sex, and PD-L1 expression. The primary endpoint was OS. At the interim analysis, with a median follow-up 9.7 months, OS was significantly improved in patients in the experimental arm (14.1 versus 10.7 months; HR 0.69; 96.71% CI: 0.55–0.87, *p* = 0.00065). The most common grade 3 to 4 IrAEs were neutropenia (7%), anaemia (6%), diarrhea (4%), increased lipase (6%), and febrile neutropenia (4%). Seven (2%) and six (2%) deaths were reported in the experimental and control group, respectively. Deaths were attributed to acute kidney failure, diarrhea, hepatotoxicity, hepatitis, pneumonitis, sepsis, and thrombocytopenia for the experimental group; and in the control group, causes included anemia, febrile neutropenia, pancytopenia, respiratory failure, and sepsis [55,56].

The 3-year update data showed the persistent benefit in OS. The median OS was 15.8 versus 11 months and median PFS was 6.4 versus 5.3 months in the experimental and the control arm, respectively. PD-L1 status did not influence the response rate; patients with PD-L1 ≥ 1% had the important finding of 28% of patients alive at 36 months, while in the PD-L1-negative population, 25% of patients were alive at 36 months. For patients with high PD-L1 expression, the median OS was 19 months [55].

### 5.5. Tremelimumab plus Durvalumab

POSEIDON, a phase 3 RCT, evaluated the effectiveness of tremelimumab plus durvalumab in 1013 patients with metastatic NSCLC without EGFR or ALK mutations regardless of histology. There were three treatment groups: (1) tremelimumab plus durvalumab plus platinum-based chemotherapy; (2) durvalumab plus platinum-based chemotherapy; and (3) platinum-based chemotherapy alone. The primary end points were PFS and OS for group 2 versus group 3. The results showed that PFS was significantly improved in patients who received durvalumab (5.5 versus 4.8 months; HR 0.74; 95% CI: 0.62–0.89, *p =* 0.0009); OS was not significantly different (5.5 versus 4.8 months; HR 0.86; 95% CI: 0.72–1.02, *p* = 0.0758). In addition, the patients in group 1 had a significantly higher PFS (6.2 vs. 4.8 months; HR 0.72; 95% CI: 0.60–0.86, *p* = 0.0003) and OS (14.0 versus 11.7 months; HR 0.77; 95% CI: 0.65–0.92; *p =* 0.0030). Regarding IRAES, grade 3–4 were presented in 51.8%, 44.6%, and 44.4% of the patients in groups 1, 2, and 3, respectively. In group 1, the most common TRAE included pneumonia (11%), anemia (5%), diarrhea (2.4%), thrombocytopenia (2.4%), pyrexia (2.4%), and febrile neutropenia (2.1%). Treatment-related deaths occurred in 3.3% (11/330) of patients in group 1 and 2.4% (8/333) in group 3; and 15.5%, 14.1%, and 9.9% discontinued treatment in groups 1, 2, and 3, respectively [54,57].

## 6. Second-Line Therapy

For cases in which a patient is not exposed to immunotherapy in the first line, single-agent is recommended as a subsequent therapy option for select patients with metastatic NSCLC. In this setting, the use of pembrolizumab is approved for patients with PD-L1 levels greater than 1%, and nivolumab or atezolizumab, regardless of PD-L1 levels (Table 4).

## 7. Atezolizumab

Two phase 2 trials, BIRCH and POPLAR, showed a benefit in OS for patients who received single-agent atezolizumab with NSCLC with high PD-L1 expression (assessed by the SP142 PD-L1 immunohistochemical assay) across multiple lines of therapy [34].

The OAK, a phase 3 RCT, assessed the efficacy and safety of atezolizumab versus docetaxel in previously treated patients with NSCLC, regardless of histology. Patients had received one to two previous chemotherapy regimens for stage IIIB or IV, except docetaxel, CD137 agonists, anti-CTLA4, anti PD-L1 or PD-1. Co-primary endpoints were OS in the ITT and PD-L1-expression population (≥1% PD-L1 on tumor cells or tumor-infiltrating immune cells). In the ITT population, OS was significantly improved in patients who received atezolizumab vs. docetaxel (13.8 versus 9.6 months; HR 0.73; 95% CI: 0.62–0.87, *p* = 0.0003) and in patients with positive PD-L1, OS was 15.7 versus 10.3 months (HR 0.74; 95% CI: 0.58–0.93, *p =* 0.0102) for patients in the atezolizumab and docetaxel groups, respectively. OS was similar in non-squamous (HR 0.73; 95% CI: 0.60–0.89) and squamous (HR 0.73; 95% CI: 0.54–0.98). Rates of Grades 3 to 4 IrAEs were reported as inferior for atezolizumab versus docetaxel (15% vs. 43%) [34,58].

The final results of the OAK trial reported a continued benefit of atezolizumab in OS, with a median OS of 13.3 versus 9.8 months, in favor of the atezolizumab group (HR 0.78, 95% CI: 0.68–0.89). The 4-year OS rates were 15.5% (12.4–18.7) and 8.7% (6.2–11.3) for atezolizumab and docetaxel, respectively. Grade 3–4 TRAE occurred in 16% of atezolizumab 4-year survivors. Atezolizumab had improved the OS benefit compared with docetaxel, regardless of PD-L1 expression and histology groups [59].

## 8. Nivolumab

CheckMate-057, a phase 3 RCT, compared nivolumab versus docetaxel as subsequent therapy for patients with metastatic non-squamous NSCLC. The primary endpoint was OS, the median OS was 12.2 months for the nivolumab group compared with 9.4 months for the docetaxel group (HR 0.73; 95% CI: 0.59–0.89, *p* = 0.002). The median duration of response was 17.2 months with nivolumab compared with 5.6 months for docetaxel. Grade 3 to 5 IrAEs were reported in 10% of the nivolumab group versus 54% in the docetaxel group. For patients without PD-L1 expression, there was no difference in OS for nivolumab versus docetaxel; however, nivolumab was associated with a longer duration of response and fewer side effects [60].

For patients with metastatic NSCLC squamous cells who progressed after the first line of CT, CheckMate-017, a phase 3 RCT, compared the efficacy and safety of nivolumab versus docetaxel. The primary endpoint was OS, median OS was 9.2 months for nivolumab compared with 6.0 months for docetaxel (HR 0.59; 95% CI: 0.44–0.79; *p* < 0.001). ORR was 20% with nivolumab compared with 9% for docetaxel (*p* = 0.008) and the median PFS was 3.5 versus 2.8 months (HR 0.62; 95% CI: 0.47 to 0.81, *p* < 0.001) for nivolumab and docetaxel, respectively. PD-L1 expression was not associated with response to nivolumab. Grade 3–4 IrAEs were reported in 7% of patients with nivolumab and 55% of patients with docetaxel. No deaths related to nivolumab were reported, but three deaths occurred in the docetaxel arm [61].

In a 5-year pooled update data of CheckMate-057 and CheckMate-017, the 5-year OS rates were 13.4% and 2.6%, respectively. Similarly, the 5-year PFS rates were 8.0% and 0% respectively. IrAEs were observed in 25.8% (8/31) of patients who received nivolumab, and within a follow-up period of 3–5 years, 7 patients experienced new IrAEs. Among these, one TRAE (3.2%) was categorized as grade 3, while there were no grade 4 IrAEs [62].

### Pembrolizumab

KEYNOTE-010, a phase 2/3 RCT, evaluated the efficacy of single-agent pembrolizumab in previously treated patients with PD-L1 positive (≥1%) advanced NSCLC, regardless of histology. Most patients were current or former smokers. There were three arms: pembrolizumab at 2 mg/kg, pembrolizumab at 10 mg/kg, and docetaxel at 75 mg/m^2^ every three weeks. The primary endpoints were OS and PFS in the total population and in patients with PD-L1 ≥ 50% of tumor cells. The median OS was 10.4 months, 12.7 months, and 5.5 months for the lower dose of pembrolizumab, the higher dose, and docetaxel, respectively. In both the low dose group (HR 0.71; 95% CI: 0.58–0.88, *p* = 0.0008) and the high dose group (HR 0.61; CI: 0.49–0.75, *p* < 0.0001), OS was significantly longer than the CT group. No significant difference was found for PFS between the three groups. For patients with at least 50% PD-L1 expression, OS in the low dose pembrolizumab (14.9 vs. 8.2 months; HR 0.54, CI: 0.38–0.77, *p* = 0.0002) and PFS (5 versus 4.1 months; HR 0.59, 95% CI: 0.44–0.78, *p* = 0.0001) groups was significantly longer. In addition, in the high dose group (OS 17.3 vs. 8.2 months; HR 0.50; 95% CI: 0.36–0.70; *p* < 0.0001) and the PFS group (5.2 versus 4.1 months; HR 0.59, CI: 0.45–0.78, *p* < 0.0001), OS was higher than the docetaxel group. Grade 3–5 TRAE were presented in 13% (43/339), 16% (55/343), and 35% (109/309) in the low dose, high dose, and docetaxel groups, respectively. Six treatment-related deaths occurred in patients receiving pembrolizumab (three at each dose) and five occurred in the docetaxel arm [63].

The 5-year efficacy and safety follow-up for the KEYNOTE-010 study reported a maintained benefit for these patients in OS, with a median follow-up of 67.4 months (60.0–77.9). The median OS was 16.9 months versus 8.2 months (HR 0.55; CI 0.44–0.69) in the PD-L1 TPS ≥ 50% group and 11.8 months versus 8.4 months (HR 0.70; CI 0.61–0.80) in the PD-L1 TPS ≥ 1% group. The 5-year OS rates for pembrolizumab versus docetaxel were 25.0% versus 8.2% in patients with PD-L1 TPS ≥ 50% and 15.6% versus 6.5% with PD-L1 TPS ≥ 1. Exploratory biomarker analysis revealed that higher tissue tumor mutational burden (≥175 mutations per exome) was associated with improved outcomes with pembrolizumab [63,64].

## 9. Adverse Effects

Unique immune-mediated adverse events (irAEs) are associated with ICIs, these are completely different to the traditional chemotherapy toxicities [65]. The spectrum of potential immune-mediated adverse events comprises cardiovascular, dermatologic, endocrine, gastrointestinal, neurologic, and pulmonary events [66].

- Cardiovascular irAEs: Myocarditis is a significant concern due to its associated high risk of mortality. Clinical presentations of ICI-induced myocarditis vary, ranging from chest pain to acute dyspnea and circulatory collapse [67]. Fatal cases have been reported even after a single dose of ipilimumab and nivolumab. Emerging data suggest that myocarditis typically occurs early in treatment, with 81% of events happening within the first four treatment cycles (around 34 days from treatment initiation) [68]. Patients undergoing ICIs should undergo a thorough cardiac assessment if any signs of cardiac insufficiency or chest discomfort arise. Baseline electrocardiograms are recommended before initiating ICI treatment, as cardiac toxicities can manifest as isolated arrhythmias. Regular monitoring of serum troponin levels is also advised due to the potential for silent myocardial injury during the course of treatment [69].
- Dermatologic irAEs: This affects a substantial portion of patients, ranging from one-third to over half of individuals. Common skin toxicities encompass rash, pruritus, and vitiligo, with similar occurrences in patients receiving either anti-CTLA-4 or anti-PD-1 antibodies [70]. The majority of lesions are maculopapular and affect less than 30% of the body surface area. Follicular, pustular, vesicular, and acneiform presentations have also been observed. ICI-induced rash often resolves within 1–2 months with effective management; however, some patients experience persistent or recurrent low-grade cutaneous toxicities after treatment completion. Severe cases, such as extensive exfoliative, ulcerative, or bullous dermatitis, are less common but can occur. Grade ≥3 cutaneous irAEs are typically seen in 2–3% of patients on ICI monotherapies and 4–10% on combination therapies. Special caution is needed when managing patients with active or past psoriasis or a family history of psoriasis [71].
- Endocrine irAEs: Hypophysitis is a rare occurrence in patients treated with anti-PD-1 antibodies. However, it is notably more common in individuals who receive ipilimumab, with an incidence ranging from 12.0% to 13.3% in real-world settings [72]. Patients experience symptoms such as fatigue, weakness, headache, visual disturbances, arterial hypotension, and nausea, and there should be heightened suspicion of hypophysitis. To diagnose hypophysitis, early performance of pituitary MRI is recommended, as is an evaluation of pituitary functional status [72,73]. In contrast to hypophysitis, thyroid dysfunction is more commonly linked with antibodies that target PD-1, rather than CTLA-4. Notably, thyroid dysfunctions have only been reported in connection with anti-PD-L1 antibodies [74]. In real-world settings, nearly 20% of patients receiving anti-PD-1 antibodies exhibit thyroid dysfunction. This typically occurs early in the treatment process, with a median onset around 6 weeks after the first infusion. Thyroid irAEs often manifest without noticeable symptoms. They may present as mild thyrotoxicosis or primary hypothyroidism resulting from destructive thyroiditis. In some cases, a less common presentation involves thyrotoxicosis tied to autoimmune thyroid disease (such as Graves’ disease) [75].
- Gastrointestinal irAEs: Colitis stands out as the most prevalent irAE observed in patients treated with ipilimumab. When undergoing endoscopic assessments, the typical findings involve a mucosa displaying widespread ulceration and edema. Interestingly, this effect can impact the entire colon rather than being confined to a specific segment [75,76]. In about a quarter of patients, there is also an associated occurrence of diffuse enteritis, a condition that can manifest even in the absence of colitis. It is essential to consider the potential presence of enteritis in cases where patients exhibit diarrhea coupled with unexplained weight loss. The incidence of colitis and/or enteritis induced by anti-PD-1 antibodies is notably lower compared to the frequency of colitis resulting from ipilimumab treatment [77]. This discrepancy underscores the potential redundancy of the PD-1 pathway in maintaining immune homeostasis within the gut [78].
- Neurologic irAEs: The spectrum involves the central and peripheral nervous system; therefore, it encompasses various conditions including myopathies, neuromuscular junction disorders, peripheral neuropathies (including axonal and demyelinating polyradiculoneuropathies), length-dependent and non-length-dependent neuropathies, asymmetric mononeuritis multiplex, cerebellar ataxia, retinopathy, bilateral internuclear ophthalmoplegia, and headache. It is important to note that ongoing research continues to expand our understanding of neurological irAEs, revealing diverse and often complex presentations [79,80].
- Pulmonary irAEs: Patients treated with anti-PD-1 antibodies have a higher likelihood of experiencing pneumonitis compared to those receiving anti-CTLA-4 antibodies. Diagnosing this life-threatening complication can be particularly challenging, especially in patients with pre-existing chronic lung diseases such as lung cancer. Radiological presentations of immune-related pneumonitis include various patterns such as cryptogenic organizing pneumonia (COP), nonspecific interstitial pneumonia, hypersensitivity pneumonitis, acute interstitial pneumonia, sarcoid-type reactions, and acute respiratory distress syndrome [81]. The management of pneumonitis involves immunosuppression with steroids and, in some cases, infliximab and/or cyclophosphamide. However, the optimal approach for frail patients requiring immunosuppression is an ongoing challenge, and research is needed to develop predictive tools for guiding treatment decisions [82].

It is essential for clinicians to recognize that, although uncommon, life-threatening irAEs continue to be documented. The ongoing accumulation of such cases in the medical literature poses a difficulty in effectively diagnosing and treating patients experiencing these events. A metanalysis was performed to evaluate the safety and effectiveness of ICIs in NSCLC. Grade 3 to 5 irAEs and health-related quality of life (HRQoL) were secondary outcomes. Data from a total of 5893 participants were included. For single agent ICIs, 5 RCT were included, with a total of 3346 participants. Patients had PD-L1 expression ≥ 50%; Grade 3–4 irAEs may be less frequent with single-agent ICI compared to platinum-based CT (RR: 0.41, 95% CI 0.33 to 0.50, I^2^ = 62%, low-certainty evidence). HRQoL data were available from only one study which suggested that single-agent ICI may improve HRQoL at 15 weeks compared to platinum-based chemotherapy (RR: 1.51, 95% CI 1.08 to 2.10, 1 RCT, 297 participants, low-certainty evidence). For double-agent ICI, irAEs were not reported according to PD-L1 expression levels. The frequency of grade 3–4 may not differ between double-ICI treatment and platinum-based CT (RR: 0.78, 95% CI 0.55 to 1.09, I^2^ = 81%, 2 RCTs, 1869 participants, low-certainty evidence). Trials did not report data on HRQoL, PFS, and ORR according to PD-L1 groups [83].

In 2022, another meta analysis evaluated serious (grade 3–5) and other (grade 1–2) irAEs. A total of 23,322 patients from 52 RCTs were included. The overall incidences of serious irAEs were 37.0% in the CT arm, 33.0% in the PD-1 arm, and 37.0% in the PD-L1 arm. In the combined groups, it was 47.0% in the PD-L1 plus CT arm, 43.0% in the PD-1 plus CTLA-4 arm, and 48.0% in the ICI plus target arm. The incidence of each serious irAE was higher in the combined groups. The ICIs groups presented a significantly higher incidence of colitis, hepatobiliary disorders, pneumonitis, and rash compared with CT. A positive correlation was observed between PFS and the occurrence of serious hepatitis (*p* < 0.0001) in the PD-L1 arm; while in the PD-1 arm it was observed with pneumonitis (*p* = 0.0049) and rash (*p* < 0.0001) [84].

In Table 5, we list the frequency of the most common irAEs [66,85,86].

## 10. Future Directions

The recognition of novel target molecules that function in separate or synergistic pathways, either in standalone treatment or combined with PD-1/PD-L1 blockade, is garnering significant attention. CD226, TGIT, and CD96 are receptors that interact with the ligand CD155 [87]. These receptors function in a competitive manner as co-stimulatory or inhibitory factors, similar to how CTLA-4, B7, and CD28 are connected in a tripartite manner. The discovery of PVRIG (also known as CD112R) and its interaction with CD112 has added intricacy to this system and provided more possibilities for therapeutic interventions [88].

In the randomized phase II CITYSCAPE trial (NCT03563716), the combination of tiragolumab (anti-TIGIT) and atezolizumab was investigated as a first-line therapy for advanced NSCLC patients with PD-L1 expression (PD-L1 ≥ 1%). The preliminary results from this trial indicated improved anti-tumor activity when compared to atezolizumab alone. TIGIT expression on T cells and natural killer cells correlates with PD-L1 expression, making the dual inhibition of these pathways potentially synergistic in mouse models [87,89]. The tiragolumab plus atezolizumab combination is undergoing further investigation in a phase III trial as a first-line therapy for advanced-stage NSCLC (NCT04294810).

In addition to anti-TIGIT, other agents that target other CD226 axis checkpoints are being investigated in combination with PD-1 inhibitors. A CD96 agent is being investigated in one first line trial and one platform trial (GSK6097608) [90]. Two PVRIG agents are in phase 1 (COM701 and GSK4381562), with others under preclinical investigation.

T-cell immunoglobulin-3 (TIM-3) has been identified as a negative regulatory molecule that plays a crucial role in maintaining immune tolerance. This protein is notably upregulated in CD8(+) T cells experiencing exhaustion, a state observed in both chronic infections and tumors [91]. TIM-3 is a type I transmembrane protein that is linked to novel cell surface molecules capable of identifying IFN-γ-producing Th1 and Tc1 cells. One of TIM-3’s primary functions is to encode the proteins TIM-1, TIM-3, and TIM-4. TIM-3 is expressed on IFN-γ-producing T cells, FoxP3+ Treg cells, macrophages, and dendritic cells [91,92].

Recent findings have highlighted TIM-3’s potential role in immune checkpoint inhibition. COSTAR Lung is a randomized, open-label design with three arms. This phase 2/3 trial aims to compare the effectiveness of different treatments in patients with advanced non-squamous NSCLC who have experienced disease progression after prior anti-PD-L1 therapy and platinum doublet-based chemotherapy. The trial involves 750 participants and the primary endpoint is OS. The three treatment arms being studied are as follows: Cobolimab (an investigational selective anti-TIM-3 monoclonal antibody) in combination with dostarlimab and docetaxel; dostarlimab in combination with docetaxel; and docetaxel alone [91,92,93].

Other innovative immunotherapy approaches include using agonists of costimulatory molecules such as 4-1BB, OX40, and TLR9 (NCT02554812), developing personalized tumor antigen-based vaccines (NCT02897765 and NCT03289962), and utilizing cytokine mimetics (NCT03520686 and NCT03625323). Adoptive cell therapy is also being explored, which has demonstrated the potential to induce profound responses in some patients with relapsed and/or refractory advanced-stage NSCLC. However, this approach is still in its early stages (NCT03308942) [94].

Other therapeutic classes are also being evaluated, including single-target and multi-targeted kinase inhibitors, PARP inhibitors, and epigenetic modifiers, in combination with anti-PD-1/PD-L1 antibodies as first-line therapies. The rationale behind these combinations is based on preclinical evidence suggesting that these agents might sensitize tumors to this class of ICIs (NCT02638090) [94,95,96].

## 11. Discussion/Practical Considerations

### 11.1. Biomarkers

Immunotherapy has become the standard therapy for metastatic lung cancer without a driver mutation. However, an important proportion of patients do not respond to such interventions. How to select the population that most benefits is an active area of research.

KRAS-mutated tumors generally exhibit a good response to ICIs. The presence of co-mutations has been described as a resistance mechanism to target treatment and has an influence on ICIs response [97]. The most common co-mutations were STK11 (encoding LKB1) and KEAP1; these had a negative influence in the response to immunotherapy, specifically in KRAS-mutated tumors. The role of STK11/LKB1 and KEAP1 mutation/Nrf2 activation as a negative prognostic/predictive biomarker is promising but requires more investigation [98].

In conclusion, the development of biomarkers (TMB, CD8, MHC-I, mIF, etc.) for the response to immunotherapy needs further study. Nowadays, PD-L1 expression could be considered as the standard for clinical practice. A biomarker for selecting patients in need of CTLA-4 blockade in addition to PD-L1 is needed [98].

### 11.2. Regimen Selection

**Figure 1:**
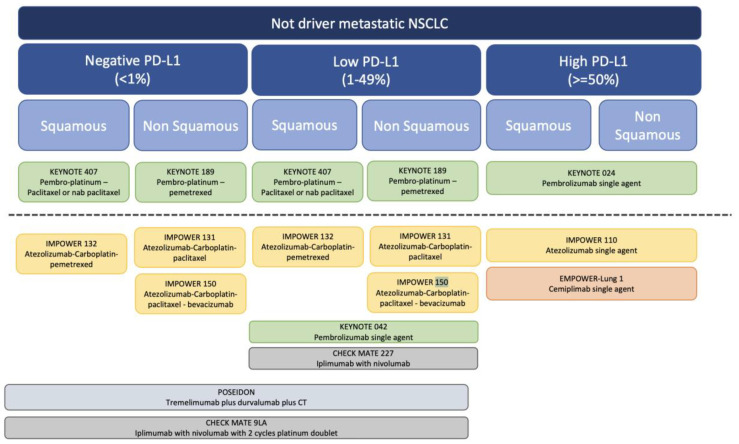
Algorithm for the Use of ICIs in not driver mNSCLC.

Currently, multiple treatment options with immunotherapy exist, including: monotherapy and combinations with chemotherapy, antiangiogenics, or other immunotherapeutic agents (Figure 1) [30].

Selecting the most appropriate treatment regimen can be a complex decision-making process, as there are several factors to consider. This decision is often influenced by individual or institutional practices due to the absence of direct comparisons between different regimens and varying toxicity profiles. In general, the choice of regimen is guided by the level of PD-L1 expression on tumor cells, the histological subtype of the cancer, relevant clinical factors, and patient preferences. Here is our suggested approach:

Tumor PD-L1 Expression: The level of PD-L1 expression on tumor cells is an important factor in selecting the appropriate regimen.

For patients with high PD-L1 expression (PD-L1 ≥ 50%), the 2-year OS was 50% with pembrolizumab (KEYNOTE-024), 52%% with pembrolizumab plus CT (KEYNOTE-189), and 48% with nivolumab plus ipilimumab (CheckMate-227). In addition, the results from KEYNOTE-407 showed a 5-year OS rate of 18.4%; and KEYNOTE-598 did not find benefit from the addition of ipilimumab to pembrolizumab in these patients. Based on these results, single agent ICIs is a reasonable choice for high PD-L1 high expressers. According to ESMO guidelines, pembrolizumab is considered the standard first-line option. However, patients with a greater burden of disease and symptoms may benefit from an upfront combination regimen with CT plus an anti-PD-1/PD-L1 antibody, given high ORRs such as 49.2% (IMpower130), 49.4% (IMpower131), 43.3% (EMPOWER-Lung 3), 47.6% (KEYNOTE-189), and 57.9% (KEYNOTE-407) [94].

For patients with low PD-L1 expression (PD-L1 1–49%), the first-line treatment options available include chemotherapy–immunotherapy combinations, pembrolizumab monotherapy, and nivolumab plus ipilimumab. In this subgroup, the combination of CT plus pembrolizumab has demonstrated significant efficacy in both histologies (KEYNOTE 189 and KEYNOTE-407) [51,94]. A similar trend towards improved OS was observed with atezolizumab plus chemotherapy, although statistical significance was not reached in comparison to chemotherapy alone. The CT–immunotherapy combinations tested in the IMpower150 and the CheckMate9LA trials have a similar benefit but with a higher risk of toxicity [44,56]. The KEYNOTE-189 and IMpower150 regimens have not been directly compared in prospective clinical trials, so the decision between them may be influenced by factors such as the patient’s medical history, potential side effects, and individual treatment goals. The choice of chemotherapy agents, as well as the preference for using [44] pemetrexed or nab-paclitaxel, can also impact the selection of the most suitable regimen.

For patients with PD-L1 negative (PD-L1 < 1%), treatment decisions can be challenging, but several options have shown efficacy. Similar to PD-L1 low expressers, pembrolizumab plus histology-selected chemotherapy may be preferable, as demonstrated in trials such as KEYNOTE-189 and KEYNOTE-407 [51,53,54]. Pembrolizumab in combination with pemetrexed has shown clear efficacy for non-squamous NSCLC, while for patients with squamous NSCLC, pembrolizumab combined with carboplatin and nab-paclitaxel has shown favorable overall survival outcomes in the PD-L1 < 1% subgroup, based on the exploratory analysis of the KEYNOTE-407 trial. The late convergence of survival curves observed in certain trials may be due to factors such as the high rate of effective crossover to second-line anti-PD-1/PD-L1 antibodies [53]. While nivolumab plus ipilimumab is not FDA approved for this specific scenario, it remains a viable option based on promising data from the PD-L1-negative subgroup of the CheckMate-227 trial. Similarly, the CheckMate-9 LA regimen involving nivolumab and ipilimumab plus carboplatin and either paclitaxel or pemetrexed has shown favorable outcomes in the PD-L1 < 1% subgroup, providing an FDA-approved and EMA-approved treatment option for both histologies [56].

Clinical Characteristics and Patient Preferences: Patient-specific factors such as smoking status, age, performance status, comorbidities, and previous treatments should be considered [94]. For example, patients with pre-existing autoimmune diseases might need closer monitoring or alternative treatment options due to the potential for exacerbation of immune-related adverse events. The patient’s preferences and goals of treatment should also be taken into account. Some patients may prioritize treatments with milder side effects, while others might be more focused on maximizing treatment efficacy.

Clinical Trials and Multidisciplinary Teams: Participation in clinical trials could provide access to novel therapies and contribute to the advancement of knowledge about ICI treatments. Treatment decisions are best made through consultation with a multidisciplinary team that includes oncologists, pathologists, radiologists, and other relevant specialists. This collaborative approach ensures a comprehensive assessment of the patient’s condition and helps in making informed treatment choices.

#### 11.2.1. Duration of Therapy

The optimal duration of anti-PD-1 and PD-L1 agents is not yet completely established. The Checkmate 153 trial, a phase IIIb/IV RCT, showed that OS is longer in patients with stable or responding disease who continued nivolumab compared with patients who stopped at 12 months (not reached vs. 32·5 months, HR 0·61, 95% CI 0.37–0.99) [99].

Additionally, excellent survival outcomes have been reported for patients who completed 24 months of nivolumab and pembrolizumab [64,65]. Moreover, a significant group of patients with disease progression after therapy completion can be successfully re-treated with an anti-PD-1/PD-L1 antibody [94].

Current data supports ICI treatment for at least 2 years for patients who maintain disease stability or response to therapy [30]. However, prospective data is needed to define the optimal duration of maintenance therapy.

#### 11.2.2. Effectiveness in Target Population

Currently, we do not have evidence that second-line or subsequent-line with immunotherapy has a benefit in patients with a driver mutation [100,101]. Indeed, studies consistently show very limited or no effectiveness of immunotherapy in this group of patients [52,54]; however, in these trials, the number of patients included in the subgroup analysis was not enough to determine statistical significance [58,60,61].

On the other hand, the concurrent use and even the sequential use (ref) of immunotherapy agents and TKIs showed significant increases in severe toxicity, such as pneumonitis and hepatitis, and no clinical benefit [102,103]. Fortunately, the development and knowledge of the genome and molecular biology has made it possible to understand the behavior of these types of tumors, and currently, there are other therapeutic alternatives for this type of population [30].

#### 11.2.3. Immunotherapy Resistance

The vast majority of patients experience disease progression. In Keynote 189, 7.5% of patients who received pembrolizumab plus CT experienced disease progression at their 5-year follow up [51]; while in KEYNOTE 407, the rate of 5-year DFS was 10.8% in the pembrolizumab arm [51,53]. Even in the case of PD-L1 TPS ≥ 50%, the majority of NSCLC patients experienced disease progression (12.8% at 5y).

The development of immunotherapy resistance in NSCLC is a complex process involving various intrinsic and extrinsic factors within the tumor. Regarding tumor-intrinsic factors, tumor cells develop mechanisms of immune evasion and heterogeneity related to immune inhibitory signals, defect antigen presentation, metabolic imbalances, and altered IFN signaling. Extrinsic factors included the tumor microenvironment (TME) or host factors. Immune cells such as tumor-associated macrophages (TAMs), B cells, natural killer (NK) cells, and T cells play significant roles in this resistance (exhaustion/dysfunction, metabolic imbalances, alternative polarization and immune suppressive signals). Host factors include gender, germline mutation, and microbiome/diet. When facing NSCLC progression after immunotherapy resistance, the choice of clinical strategies should be guided by the mode of progression [51,104,105].

Resistance to immunotherapy in metastatic NSCLC has different clinical scenarios: Innate-primary resistance/hyper progression, acquired/adaptive resistance after partial response/stable disease, and progression after a durable benefit (oligo progression and systemic progression) [106]. Oligo progression refers to a scenario in which only a few isolated lesions show progression, while the majority of the disease remains stable. Systemic or multiple progression indicates widespread disease progression affecting multiple sites. Treatment decisions should consider these patterns to determine the appropriate next steps [105].

Optimizing combined therapy is a key strategy for addressing immunotherapy resistance in NSCLC. Understanding how to best combine different therapeutic approaches can enhance the effectiveness of treatment. Additionally, strategies should be explored to reprogram immune cells that infiltrate the tumor microenvironment. This involves modifying the behavior of these immune cells to better target and attack tumor cells. It is important to adapt these strategies to the genetic diversity of tumor cells and to reshape the tumor microenvironment in a timely manner during the course of antitumor treatments.

## 12. Conclusions

The field of immunotherapies has revolutionized the treatment of mNSCLC and has emerged as an essential part of first-line therapy, leading to significant improvements in long-term survival. Although we do not yet have an ideal biomarker that allows us to determine which immunotherapy would best suit an individual case, there is no doubt that PD-L1 has great clinical utility so far. Nonetheless, the future of immunotherapy selection is likely to involve the integration of various novel biomarkers to enable a more personalized approach. This could include a deeper exploration of genomic factors, such as the predictive role of specific genetic variants (somatic or germline), as well as a more intricate understanding of the dynamics within the tumor microenvironment.

When it comes to selecting the most appropriate treatment (Figure 1), multiple options exist, and given the lack of comparative trials, deciding which treatment should be used is increasingly complex. Based on the PD-L1 status, the recommended approach for those with high PD-L1 expression is single-agent ICIs, while a combination regimen (CT plus ICI, CT plus antiangiogenic plus ICI, CT plus ICI+ ICI) is favored for patients with low or negative PDL1 expression. These combination therapies have exhibited comparable effectiveness, with slightly higher response rates noted for pembrolizumab. Consequently, this has positioned pembrolizumab as the primary choice according to leading guidelines. Nevertheless, the choice of treatment should be personalized for each patient, and it is crucial to consider other factors such as histology, some clinical characteristics, the toxicity profile, and accessibility; which is particularly relevant given the current healthcare systems. In addition, future research should emphasize the development of more accurate methods for predicting which patients will respond favorably to first-line immunotherapies and which might develop resistance.

## Figures and Tables

**Table 1:** ICI approved for treatment in metastatic NSCLC.

Drug	Type	Indications
Atezolizumab	Anti-PD-L1	First-line treatment (*, **): - Monotherapy for patients with mNSCLC without EGFR or ALK genomic tumour aberrations and PD-L1-stained ≥ 50% of tumour cells or PD-L1-stained tumour-infiltrating immune cells ≥ 10%. - In combination with bevacizumab and chemotherapy (platinum- based + paclitaxel/nab-paclitaxel) for patients with metastatic non-squamous NSCLC without EGFR or ALK aberrations and regardless of PD-L1 status. - In combination with chemotherapy (platinum- based + paclitaxel/nab-paclitaxel/pemetrexed) for patients with metastatic non-squamous NSCLC without EGFR or ALK aberrations and regardless of PD-L1 status. Subsequent line monotherapy for the treatment of patients with mNSCLC who have disease progression during or following platinum-containing chemotherapy regardles histology and PD-L1 status (*, **).
Cemiplimab	Anti-PD1	First-line treatment (*, **): - Monotherapy for patients with mNSCLC with no EGFR, ALK, or ROS1 aberrations and with high PD-L1 expression [Tumour Proportion Score (TPS) ≥ 50%]. - In combination with chemotherapy for patients with mNSCLC without EGFR, ALK, or ROS1 aberrations, regardless of histology and PD-L1 status.
Nivolumab	Anti-PD1	Subsequent line monotherapy for the treatment mNSCLC who has been progressed after platinum-based chemotherapy. Patients with EGFR or ALK aberrations must have disease progression at least one target therapy (*, **).
Nivolumab/Ipililumab	Anti-PD1/Anti-CTLA4	First-line treatment (*, **): - In combination for patients with mNSCLC expressing PD-L1 ≥ 1%, with no EGFR or ALK genomic tumour aberrations. - In combination with platinum-based chemotherapy for two cycles in adults, whose tumours have no EGFR mutation or ALK translocation, regardless of PDL-1 expression.
Pembrolizumab	Anti-PD1	First-line treatment (*, **): - Single agent for patients with NSCLC expressing PD-L1 (TPS) ≥ 1% with no EGFR or ALK genomic tumour aberrations. - In combination with pemetrexed and platinum chemotherapy for patients with metastatic non-squamous NSCLC, without EGFR or ALK genomic tumour aberrations. - In combination with carboplatin and either paclitaxel or nab-paclitaxel for patients with metastatic squamous NSCLC. Subsequent-line treatment (*, **): - Single agent patients with mNSCLC with PD-L1 (TPS ≥ 1%), who had progression on or after platinum-containing chemotherapy. Patients with EGFR or ALK aberrations must have disease progression with at least one target therapy.
Tremelimumab/Durvalumab	Anti-CTLA4/Anti-PD-L1	First-line treatment in combination with platinum-based chemotherapy for the treatment of patients with mNSCLC without EGFR or ALK genomic tumour aberrations, regardless of histology.

(*) Approved by U.S. Food and Drug Administration (FDA). (**) Approved by European Medicines Agency (EMA).

**Table 2:** Single agents ICI for treatment of NSCLC at first line.

ICIs	Trial	Population	Primary Endpoint	ORR	PFS	OS	5y-ORR	5y-PFS	5y-OS
Atezolizumab	IMpower 110	PD-L1 ≥ 50% or IC ≥10%; squamous and non-squamous histology	OS	38.3 vs. 28.6%	8.1 vs. 5.0 months	20.2 vs. 13.1 months	40.2%	8.2 months	20.2 vs. 14.7 months
Cemiplimab	EMPOWER-Lung 1	PD-L1 ≥ 50%; squamous and non-squamous histology	OS and PFS	39.0 vs. 20%	8.2 vs. 5.7 months	NR vs. 14.2 months	46.5 vs. 21.0% *	8.1 vs. 5.3 months *	26.1 months *
Nivolumab plus ipilimumab	CheckMate 227	PD-L1 ≥ 1%; squamous and non-squamous histology	OS	35.9 vs. 30.0%	5.1 vs. 5.6 months	17.1 vs. 14.9 months			24%
Pembrolizumab	KEYNOTE-024	PD-L1 ≥ 50%; squamous and non-squamous histology	PFS	45%	10.3 months	26.3 months (80.2%).	46.1 vs. 31.1%	7.7 vs. 5.5 months	26.3 vs. 13.4 months (31.9%)
	KEYNOTE-042	PD-L1 ≥ 1%; squamous and non-squamous histology	OS	27 vs. 27%	5.4 vs. 6.5 months	16.7 vs. 12.1 months	27.3%	5.6 months	16.4 months

Abbreviations: Objective response rate (ORR), Progression-free survival (PFS), Overall survival (OS). * 3y of follow-up.

**Table 3:** Combined agents ICI for treatment of NSCLC at first line.

ICIs	Trial	Population	Primary Endpoint	ORR	PFS	OS	5y-ORR	5y-PFS	5y-OS
Atezolizumab plus Bevacizumab plus CT	IMpower150	Any PD-L1 and Non-squamous histology	PFS and OS	63.5% (ABCP) vs. 48.0% (BCP)	8.3 vs. 6.8 months ABCP vs. BCP HR 0.62 (95% CI 0.52–0.74)	19.5 vs. 14.7 months ABCP vs. BCP: HR 0.78 (95% CI 0.64–0.96)		8.4 vs. 6.8 months ABCP vs. BCP HR 0.57 (95% CI 0.48–0.67)	19.5 vs. 14.7 months HR 0.80 (95% CI 0.67–0.95)
Atezolizumab plus platinum plus paclitaxel/nab paclitaxel	IMpower130	Any PD-L1 and Non-squamous histology	PFS and OS	49.2% vs. 31.9%	7.0 versus 5.5 months (HR 0.64; 95% CI 0.54–0.77)	18.6 versus 13.9 months (HR 0.79; 95% CI 0·64–0·98)	-	-	-
Atezolizumab plus platinum plus paclitaxel/nab paclitaxel	IMpower131	Any PD-L1 and squamous histology	PFS and OS	49.4% vs. 41.3%	6.3 vs. 5.6 months HR 0.71 (95% CI 0.60–0.85)	14.2 versus 13.5 months (HR 0.88; 95% CI 0.73–1.05)	-	-	-
Atezolizumab plus platinum plus pemetrexed	IMpower132	Any PD-L1 and Non-squamous histology	PFS and OS	47% vs. 32%	7.6 versus 5.2 months; HR 0.60, 95% CI 0.49–0.72	18.1 versus 13.6 months; HR 0.81, 95% CI 0.64–1.03	-	-	17.5 vs. 13.6 months *; HR 0.86 (0.71–1.06)
Cemiplimab plus platinum-doublet chemotherapy	EMPOWER-Lung 3	Any PD-L1; squamous and Non-squamous histology	OS	43.3% vs. 22.7%	8.2 vs. 5.0 months HR = 0.56; 95% CI, 0.44–0.70	21.9 vs. 13.9 months; HR 0.71; 95% CI, 0.53–0.93	43.6% versus 22.1%	8.2 months versus 5.5 months (HR 0.55, 95% CI 0.44–0.68	21.1 versus 12.9 months; HR 0.65, 95% CI 0.51–0.82
Nivolumab plus ipilimumab and 2 cycles of platinum-doublet chemotherapy	CheckMate 9LA	Any PD-L1; squamous and Non-squamous histology	OS	37.7% vs. 25.1%	6.8 vs. 5.0 months HR 0.70 [97·48% CI 0.57–0.86	14.1 versus 10.7 months; HR 0.69; 96.71% CI 0.55–0.87	38% vs. 25% *	6.4 versus 5.3 months *	15.8 versus 11 months *; HR 0.74, 95% CI 0.62–0.87
Pembrolizumab plus platinum (carboplatin or cisplatin) plus pemetrexed	KEYNOTE-189	Any PD-L1 and Non-squamous histology	PFS and OS	47.6% vs. 18.9%	8.8 vs. 4.9 months HR 0.52 (95% CI 0.43–0.64)	NR vs. 11.3 months; HR 0.49; 95% CI 0.38–0.64	48.3% vs. 19.9%	9.0 versus 4.9 months; HR 0.5; 95% CI 0.42–0.60	22.0 versus 10.6 months; HR 0.6; 95% CI 0.50–0.72
Pembrolizumab plus platinum (carboplatin or cisplatin) plus Paclitaxel or nab-paclitaxel	KEYNOTE-407	Any PD-L1 and squamous histology	PFS and OS	57.9% vs. 38.4%	6.4 versus 4.8 months; HR 56; 95% CI 0.45–0.70	15.9 months and 11.3 months HR 0.64; 95% CI 0.49–0.85	66.2% vs. 38.8%	8 versus 5.1 months; HR 0.62; CI 0.52–0.74	17.2 versus 11.6 months HR 0.71; 95% CI 0.59–0.85
Tremelimumab plus durvalumab plus CT	POSEIDON	Any PD-L1; squamous and Non-squamous histology	PFS and OS	46.3% vs. 33.4%	6.2 v 4.8 months; HR 0.72; 95% CI 0.60–0.86	14.0 versus 11.7 months; HR 0.77; 95% CI 0.65–0.92	-	-	-

Abbreviations: Immune checkpoints inhibitors (ICIs), Objective response rate (ORR), Progression-free survival (PFS), Overall survival (OS). * 3y of follow-up.

**Table 4:** Second-line ICI treatments.

ICIs	Trial	Population	Primary Endpoint	ORR	PFS	OS
Atezolizumab	OAK	Patients with NSCLC, any histology, who had received one or two previous chemotherapy regimens for stage IIIB or IV, except docetaxel, CD137 agonists, anti-CTLA4, anti PD-L1 or PD-1.	OS	14% vs. 13%	2.8 VS. 4.0 months; HR 0.95 95% CI 0.82–1.10	13.8 vs. 9.6 months; HR 0.73; 95% CI 0.62–0.87. 4y rate: 15.5% vs. 8.7%.
Nivolumab	CheckMate-057	Patients with metastatic non-squamous NSCLC who progressed after the first line with platinum-based doublet CT.	OS	19% vs. 12%	2.3 vs. 4.7 months (HR 0.92; 95% CI 0.77–1.11	12.2 vs. 9.4 months (HR 0.73; 95% CI 0.59–0.89
	CheckMate-017	Patients with metastatic squamous NSCLC who progressed after the first line with platinum-based doublet CT.	OS	20% vs. 9%	3.5 versus 2.8 months (HR 0.62; 95% CI 0.47–0.81	9.2 vs. 6.0 months HR 0.59; 95% CI 0.44–0.79
Pembrolizumab	KEYNOTE-010	Previously treated patients with PD-L1 positive (≥1%) advanced NSCLC, regardless histology	PFS and OS	18% vs. 9%	4.0 versus 4.0 months (HR 0·79, 0.66–0.94	12.7 vs. 5.5 months HR 0.71; 95% CI 0.58–0.88 5y: 11.8 months versus 8.4 months (HR 0.70; CI 0.61–0.80)

Abbreviations: Immunocheckpoints inhibitors (ICIs), Objective response rate (ORR), Progression-free survival (PFS), Overall survival (OS).

**Table 5:** Frequency of reported Immune-related adverse events.

Drug	Dose	Diarrhea	Colitis	Pulmonary	Rash	Neurological	Endocrinopathy	Hepatic	Renal
Atezolizumab Trials									
Impower 110	1200 mg 3-weekly	-	-	4.9%	-	-	-	-	-
Impower 150	1200 mg 3-weekly	20.6%	-	-	13.3%	49.4% (neuropathy included)	-	-	
Oak	1200 mg 3-weekly	15.4%	0.3%	1%	-	-	-	0.3%	-
Cemiplimab Trials									
Empower-Lung 1	**350 mg** 3-weekly	**5%**	**1%**	**6%**	**5%**	**3%**	-	**6%**	**1%**
Empower-Lung 1	**350 mg** 3-weekly	**10.6%**	-	**12.5%**	-	-	-	**30%**	-
Nivolumab Trials									
CheckMate 057	3 mg/kg, 2-weekly	8%	1%	4.9%	9%	0.3%	10.5%	10.8%	2%
Nivolumab/Ipililumab									
Check Mate 227	**1 mg/kg** **6-weekly** **ipilimumab** **plus 3 mg/kg** **2-weekly** **nivolumab** **(576)**	16.3%	1%	3%	**16.7%**	-	**12.3%**	**3.5%**	-
Pembrolizumab Trials									
Keynote-054	200 mg, 3-weekly	19.1%	3.7%	4.7%	16.1%	-	23.4%	1.8%	0.4%
Keynote-010	10 mg/kg, 3-weekly	6%	1%	4%	13%	-	16.5%	1%	-
**Keynote-189**	200 mg, 3-weekly	-	**2.2%**	**4.4%**	**2%**	-	**12.8%**	-	**1.7%**
Keynote-042	200 mg, 3-weekly	**5%**	**1%**	**8%**	**7%**	**1%**	**18%**	**7%**	**<1%**
Tremelimumab/Durvalumab									
**Poseidon**		**13.9%**	**3.9%**	**3.6%**	**3.9%**	-	-	-	-
